# Control of Ventricular Ciliary Beating by the Melanin Concentrating Hormone-Expressing Neurons of the Lateral Hypothalamus: A Functional Imaging Survey

**DOI:** 10.3389/fendo.2013.00182

**Published:** 2013-11-25

**Authors:** Grégory Conductier, Agnès O. Martin, Pierre-Yves Risold, Sonia Jego, Raphaël Lavoie, Chrystel Lafont, Patrice Mollard, Antoine Adamantidis, Jean-Louis Nahon

**Affiliations:** ^1^UMR7275, Institut de Pharmacologie Moléculaire et Cellulaire, Centre National de la Recherche Scientifique, Valbonne, France; ^2^University of Nice Sophia Antipolis, Nice, France; ^3^UMR5203, Institut de Génomique Fonctionnelle, Centre National de la Recherche Scientifique, Montpellier, France; ^4^U661, INSERM, Montpellier, France; ^5^UMR-5203, Universités de Montpellier 1 & 2, Montpellier, France; ^6^Laboratoire d’Histologie, IFR 133, Faculté de Médecine et de Pharmacie, Besançon, France; ^7^Douglas Mental Health University Institute, Montreal, QC, Canada; ^8^Station de Primatologie, UPS 846, Centre National de la Recherche Scientifique, Rousset sur Arc, France

**Keywords:** MCH, MCHR1, non-neuronal function, cilia, CSF flow

## Abstract

The cyclic peptide Melanin Concentrating Hormone (MCH) is known to control a large number of brain functions in mammals such as food intake and metabolism, stress response, anxiety, sleep/wake cycle, memory, and reward. Based on neuro-anatomical and electrophysiological studies these functions were attributed to neuronal circuits expressing MCHR1, the single MCH receptor in rodents. In complement to our recently published work (1) we provided here new data regarding the action of MCH on ependymocytes in the mouse brain. First, we establish that MCHR1 mRNA is expressed in the ependymal cells of the third ventricle epithelium. Second, we demonstrated a tonic control of MCH-expressing neurons on ependymal cilia beat frequency using *in vitro* optogenics. Finally, we performed *in vivo* measurements of CSF flow using fluorescent micro-beads in wild-type and MCHR1-knockout mice. Collectively, our results demonstrated that MCH-expressing neurons modulate ciliary beating of ependymal cells at the third ventricle and could contribute to maintain cerebro-spinal fluid homeostasis.

## Introduction

First identified in the early 80s from chum salmon pituitaries, the melanin concentrating hormone (MCH) draw its name from its capability to induced the concentration of melanin in the skin melanophores (2). However, this function seems to be restricted to teleosts [reviewed in Ref. (3)]. In contrast with high MCH structural conservation, the neuronal distribution appears quite different, reflecting evolutionary changes in the prosencephalon across vertebrate species (4). In mammals, this cyclic peptide is mainly expressed in neurons of the lateral hypothalamic area (LHA), projecting widely throughout the brain (5); reviewed in Ref. (6). Accordingly, MCH is involved in a broad spectrum of cerebral functions [for recent reviews, see Ref. (7, 8)]. Nevertheless, all of these seem to converge to the adaptation of global physiologic state to metabolic needs by promoting memory processes and reward pathways activation on one hand and by decreasing arousal and thermogenesis on the other hand. Activation of these cognitive and neuroendocrine networks leads to an increase in food intake and energy storage, respectively [reviewed in Ref. (9, 10)].

The structure of the *Pmch* gene locus appears to be complex and sense/antisense transcripts could generate different protein-derivatives. Indeed, the precursor ppMCH may be processed mainly, but not exclusively, in two different peptides (MCH and NEI) in the brain and in several intermediates, including the dipeptide MCH-NEI, in peripheral organs (11–14). An additional protein, named MGOP, may be produced by an alternative splicing of the *Pmch* gene primary transcript in all cells producing MCH (15, 16). Finally a set of proteins, involved in DNA repair, may be synthesized by expression of the *AROM/PARI* gene located on the complementary strand overlapping the *Pmch* gene (8, 17). Based on this disparity in gene-products expression, it is quite difficult to associate a single molecular substrate responsible to the wide phenotypic changes observed in *Pmch* gene KO mice in which the full exon-intron sequences of the *Pmch* gene as well as the 3′UTR region of spliced AROM/PARI gene transcripts were deleted. Meanwhile, the issue of developmental compensation (or adaptation) in these genetic models of *Pmch* gene inactivation should also be considered [see Ref. (9) for discussion of this point].

Efforts to identify the MCH receptor initially led to the discovery of a spliced variant of the seven-transmembrane G-coupled protein named SLC-1 (18) as a cognate MCH receptor and thereafter referred to as MCHR1 (19–23). MCHR1 is widely localized in brain regions involved in the control of neuroendocrine, reward, motivational, and cognitive aspects of feeding behavior (9, 10, 24–26). Interestingly, MCHR1-deficient mice are lean due to hyperactivity and increased metabolism (27). A second MCH receptor, named here MCHR2, was identified and characterized in human tissues and cell lines (27–33). This MCH receptor displayed a brain distribution that overlapped partially with that of MCHR1 in the primate and fish brain (32, 34). However, MCHR2 is lacking in rat and mouse genomes (35). Furthermore, in contrast to MCHR1 that signals to either Gai or Gaq, depending on the transfected or native cell systems, MCHR2 signaling operates apparently exclusively through Gaq protein [our unpublished data; reviewed in Ref. (35–37)].

Based on neuro-anatomical mapping and electrophysiological data, it was assumed that synaptic transmission represents the main mode of action of MCH in the brain. However, non-neuronal intercellular communication or “volume” transmission may also be involved but evidence were lacking. In a recently published study (1), we mapped numerous MCH fibers in close vicinity to MCHR1 expressed into ependymocytes of the ventral part of the third ventricle (3V). Developing new techniques to measure and analyze the ependymal cilia beat frequency (CBF) in acute mouse brain slice preparations, we also showed that the CBF is increased by MCH application or LHA stimulation, an effect blocked by a selective MCHR1 antagonist and absent in MCHR1-knockout (MCHR1-KO) mice. In addition, using *in vivo* brain MRI, we demonstrated that the volume of both the lateral and third ventricles is increased in MCHR1-KO mice compared to their wild-type (WT) littermates. Thus, our study revealed a previously unknown function of the MCH/MCHR1 signaling system in non-neuronal cells. Here, we first demonstrated MCH mRNA expression in the ventral 3V ependymal cells isolated by laser-capture and *in situ* hybridization. We then extended our previous work, by using *in vitro* optogenetic activation or inhibition of MCH neurons. Finally, we investigated *in vivo* tracking of fluorescent micro-beads through the 3V in WT and MCHR1-KO mice. Collectively, we demonstrate a dynamic control of MCH neurons on spontaneous CBF of MCHR1 mRNA-expressing ependymal cells and discuss the current strategies for measuring CSF flows in small animal models.

## Materials and Methods

### Animals

The experiments were conducted with male C57BL/6J mice (for laser-captured cell mapping, *in situ* hybridization and cellular optogenetic measurements) and female KO MCHR1 mice (*in vivo* CSF flow experiments) of 10–12 weeks of age. The animals were obtained from heterozygous breeding in the local animal facilities and maintained on a 12-h dark/light cycle (7 a.m./7 p.m.) with food and water *ad libitum*. The MCHR1-KO mice were established as previously described (38).

All the protocols were carried out in accordance with French ethical guidelines for laboratory animals (Agreement N°75–178, 05/16/2000) and were approved by the IPMC care committee. Attention was paid to use only the number of animals requested and necessary to generate reproducible results.

### Laser micro-dissection of third ventricle epithelium

After decapitation, each brain (*n* = 2) was dissected out in <2 min and immediately frozen at −80°C using a Snapfrost (Alphelys, France). Sections (10 μm thick) were cut on a cryostat (Microm HM 560; object holder and chamber were kept at −21°C). Eight sections passing through the posterior hypothalamus were collected on pen membrane slides. Slides, continuously maintained on dry ice, were dehydrated in three baths of increasing ethanol baths (70, 95, and 100%) and two baths of fresh xylene (Roth, France) for 5 min each. Sections were air dried and kept in the vacuum of a dessicator until dissection.

Dissections were performed using a PixCell^®^ (Arcturus Engineering) with CapSure^®^ HS LCM caps. The dissection time never exceeded 20 min/slide, starting from when the slide was removed from the dessicator. Laser parameters were calibrated for each dissection by measuring the impact of shots on the membrane of the slide adjacent to the tissue. The area of interest was then dissected and laser-captured using UV laser to cut the tissue and IR laser to capture the sample. Four samples were collected per cap (micro-dissection of two slides in <40 min in total) and only one cap per brain. As soon as the fourth sample was obtained, the cap was examined under the microscope to ensure the absence of unwanted debris. The sample lysis and the RNA extraction were performed using the RNAqueous^®^-Micro Kit (Ambion, France) following the manufacturer’s instructions. The quality of the samples was finally evaluated with the Agilent 2100 Bioanalyzer (Agilent Technology). A rin of 6.1 was twice obtained.

After reverse transcription (Superscript III, Invitrogen), cDNA corresponding to 1 ng of RNA was used as input in a PCR reaction (GoTaq Green MasterMix, Promega, Charbonnières, France) for MCHR1 and HPRT as positive control (MCHR1 F: 5′-GCTCTATGCCAGGCTTATCC-3′, MCHR1 R: CAGCTGTCTGAGCATTGCTG-3′, amplicon size: 494 bp; HPRT F: CTCCGGAAAGCAGTGAGGTAAG, HPRT R: GGAGGGAGAAAAATGCGGAGTG, amplicon size: 306 bp). Sample for which reverse transcriptase was omitted was used as negative control. PCR protocol used was designed as follow: initial denaturation: 95°C, 5 min follow by 40 cycles composed of 95°C, 30 s, 58°C, 30 s, 72°C, 1 min, and a final elongation for 7 min at 72°C.

### *In situ* hybridization

Frozen sections were post-fixed in 4% paraformaldehyde in 0.1 M phosphate buffer and digested with proteinase K (1 μg/mL, Roche) for 30 min at 37°C. Slides were incubated for 8 min in 0.1 M triethanolamine (TEA), pH 8.0, and then for 5 min at room temperature in 100 mL 0.1 M TEA + 500 μL acetic anhydride followed by a decarboxylation in active diethyl pyrocarbonate (DEPC).

Sections were then rinsed briefly with 5× standard citrate sodium (SSC) buffer then incubated for 2 h in prehybridization buffer at 56°C. After rinsing in 0.2× SSC, the sections were incubated overnight at 56°C, in humid chambers, with 50 μL hybridization buffer containing 5% Denhardt’s and 50 ng labeled RNA probes. After rinsing with 5× SSC, sections were incubated successively in 2× SSC at 56°C (1 h 30 min) and 0.2× at room temperature (5 min). They were incubated in anti-DIG Fab fragments conjugated to alkaline phosphatase (1/1300, overnight) and revealed with enzyme substrate NBT-BCIP (overnight, at room temperature).

Two MCHR1 RNA probes were used; one probe was kindly provided by Drs Civelli and Chung (University of California, Irvine, CA, USA) and one made by reverse transcription/polymerase chain reaction from mouse genomic DNA. Control hybridization, including hybridization with sense DIG-labeled riboprobes was realized and did not reveal any signal.

### Measures of cilia beat frequency using MCH neuron-specific optogenetic excitation or inhibition

We have previously shown that electrical stimulation of the LHA induced an increase in the CBF in the 3V (1). To further extend and improve the specificity of the response, we used new models allowing the optogenetic control of MCH neurons activity. All procedures and controls were previously published in (39). Briefly, using cre-dependent Ef1a-DIO-ChETA-EYFP AAV mediated transduction, the fast mutant of the light-activated Channel rhodopsin-2 (ChETA) or the chloride pump halorhodopsin (NpHR) was specifically expressed in MCH neurons of two different groups of 3 week-old mice. Four weeks after stereotactic injection of the AAV vectors in the LHA (AP: −1.45 mm, ML: ±1 mm, DV: −5.5 mm), brain slices were made and recordings were made in CBF as described elsewhere (1) before, during, and after stimulation of ChETA-expressing MCH neurons (473 nm, stimulation frequency: 1, 5, 10, 20, and 40 Hz, pulse length: 10 ms, total stimulation duration: 3 min) or NpHR (590 nm, continuous stimulation during 8.5 s each 10 s, 5 min). For each slice and area of recording, the instantaneous CBF was calculated. All samples in which basal CBF was out of a range comprised between 5 and 20 Hz (considered as the natural basal frequency in healthy slices) were not included. The mean of the basal frequency during the first 5 min of recordings were used as a baseline for normalization of the experimental values. Results are expressed as the percentile variation of this baseline.

### Measure of CSF flow index using fluorescent micro-beads

Littermate controls and female MCHR1-KO mice (aged 12 weeks old) were anesthetized by intraperitoneal injection of ketamine hydrochloride 50 mg/kg and xylazine 10 mg/kg and placed in a stereotaxic frame tip of a 26 gage needle was brought to the following coordinates relative to the bregma: 1.75 mm posterior, 2.5 mm ventral, and 0 mm right and left. About 10 μL of polystyrene beads (diameter 3 μm (sigma L4530) dilution 1:4 in 0.09% NaCl) was injected in the third ventricle.

Fibered confocal fluorescence microscopy (FCFM) (CellviZio; Mauna Kea Technologies, Paris, France) imaging was used to visualize the *in vivo* movement of the polystyrene beads in the CSF flux. FCFM provides an easy access to these regions of interest with low disturbance of brain structure (40, 41). Small-diameter fiber-optic probe consisting of tens of thousands of fibers was implanted in the brain of the mice and connected to a Laser scanning unit LSU-488 (FibroScan) that uses a laser source with a wavelength of 488 nm. We used a MiniZ probe of 300 μm diameter with a working distance of 70 μm. The probe was stereotaxically lowered in the third ventricle at 2.5 mm ventral. Sensitive, single-pixel detection of fluorescence stimulated by the photodiode laser pulse through each fiber element, combined with the high scan rate allows the visualization of beads movements. Four acquisition sessions of at least 10 min was recorded for each animal at a frame rate of 11 frames/s.

### Statistics

#### Variation in the CBF

Statistics were performed using Prism software (Graphpad Inc., La Jolla, CA, USA). The global mean for grouped time points (baseline, stimulation, and recovery) were compared Using One way ANOVA followed by Bonferroni’s multiple comparison test (BMCT). N = number of mice, n = number of slices, *n* = number of cells considered. *p* Value <0.05 were considered significant.

#### Measure of CSF flow index using fluorescent micro-beads

Movies were visualized on ImageCell™ viewer. The speed of the beads was analyzed by tracking 10 beads/10 min films. The mean speed for an animal was the mean of the four films speeds. The movies displaying significant modifications of the speed over time were excluded as probably corresponding to pressure due to probe positioning or blood clot.

## Results

### Laser micro-dissection of third ventricle epithelium and *in situ* hybridization

**Figure 1:**
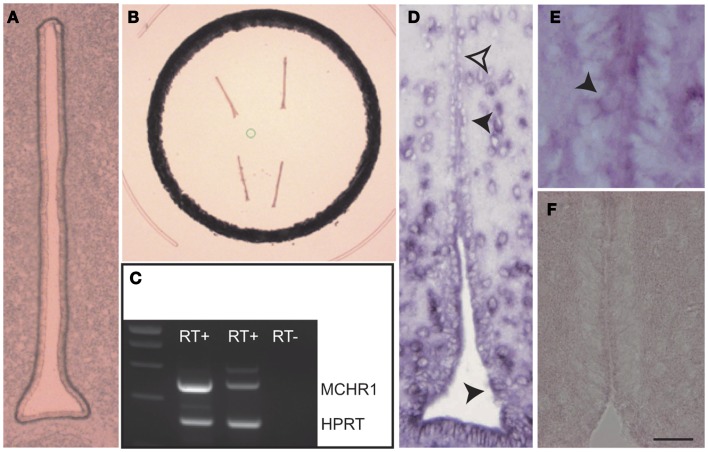
**(A)** Photomicrograph of the ventricular epithelium before laser micro-dissection. **(B)** Photomicrograph of the ventricular epithelium after laser micro-dissection. **(C)** Illustration of the RT-PCR showing the presence of MCHR1 mRNA in the ventricular epithelium. RT^+^: after reverse transcription, RT^−^: negative control of the reverse transcription. HPRT: positive control. **(D)** Photomicrograph to illustrate the distribution of the MCHR1 *in situ* hybridization signal in the periventricular hypothalamus. Ependymocytes expressed the *in situ* signal (black arrowheads), but not all of them were labeled (open arrowhead). **(E)** High magnification to illustrate cytoplasmic expression of MCHR1 mRNA in discrete ependymal cells. **(F)** A negative control using sense MCHR1 gene probe. Scale bar = 25 μm in **(A)**; Scale bar = 20 μm in (D); Scale bar = 10 μm in **(E)** and **(F)**.

As illustrated in Figures 1A,B; ependymal cell layer was carefully dissected and used for RNA extraction. RT-PCR results indicate that mRNA coding for MCHR1 were present in the 3V epithelium (Figure 1C). This was further confirmed by *in situ* hybridization with two specific probes recognizing MCHR1 mRNA. Indeed, numerous (but not all; see open arrowhead) ependymocytes were labeled with antisense probe (Figure 1D), within the cytoplasm (Figure 1E), while sense probe did not stain any cell types (Figure 1F). These results are in agreement with our immunohistochemical study (1) and recent data from Maratos-Flier’s lab using a *MCHR1-cre/tdTomato* mouse strain (26).

### MCH neuron-specific optogenetic tools

The ChETA-NpHR system was used to dissect MCH neuronal circuitry reaching 3V ependymal cells and controlling CBF.

**Figure 2:**
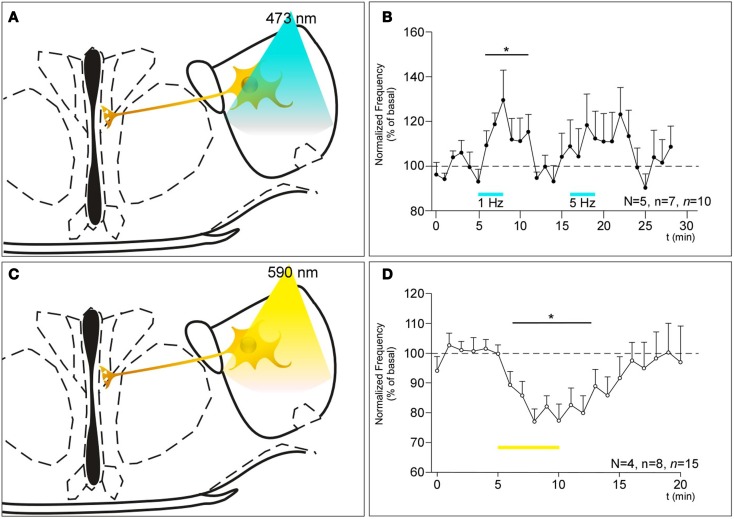
**(A,C)** Schematics showing the projection of MCH neurons expressing ChETA **(A)** or NpHR **(C)** from the LHA to the ventricular epithelium and their optogenetic stimulation paradigm. **(B)** Consequences of the stimulation of ChETA in MCH neurons, by light pulses of 10 ms at a rate of 1 and 5 Hz as shown by the bars, on the CBF recorded in the ventral 3V expressed as a percentage of the basal frequency. N: number of animals, n: numbers of slices, *n*: number of recording area. *p < 0.05 **(D)** Consequences of the stimulation of NpHR in MCH neurons as shown by the bars, in MCH neurons on the CBF recorded in the ventral 3V. N: number of animals, n: numbers of slices, *n*: number of recording area.

The stimulation of ChETA-expressing MCH neurons in the LHA induces an increase in the CBF reaching 134% of the basal value (Figures 2A,B; 1 Hz, N = 5, n = 7, *n* = 10, *F*_5.069, 29_ = 0.0131, BMCT: baseline vs. ChETA *t* = 2.896, *p* < 0.05). After 10 min recovery, the subsequent stimulation at 5 Hz tended to increase the CBF but did not reach the significance level (Figure 2B; 5 Hz, N = 5, n = 7, *n* = 10). For higher frequencies, no effect was observed (not shown, see Discussion). On the other hand, the stimulation of NpHR induced a marked decrease in the CBF reaching 76% of the basal (Figures 2C,D; N = 4, n = 8, *n* = 15, *F*_4.616, 44_ = 0.0154, BMCT: baseline vs. NpHR *t* = 3.027, *p* < 0.05).

### Measure of CSF flow index using fluorescent micro-beads

**Figure 3:**
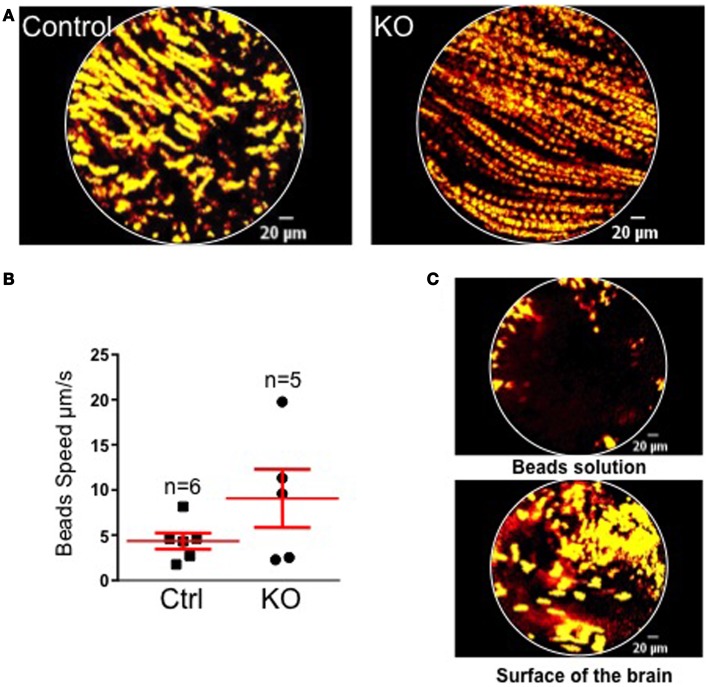
**(A)** Projections of 10 s acquisition movies illustrating the fluorescent beads movement in the CSF of control (left) and KO MCHR1 (right) animals. In both cases, beads displayed a continuous and orientated movement lasting for at least the recording time. **(B)** Mean speed of the beads in control and KO MCHR1 animals. **(C)** Negative controls. Visualizations of fluorescents beads in solution (top) and at the surface of the living brain (bottom). No orientated movements were detected.

In order to address the physiological relevance of MCH-driven ciliary beating regulation, we conducted *in vivo* flow measure. FCFM imaging was used to visualize the movement of the polystyrene beads in the CSF flux *in vivo* in groups of WT and KO MCHR1 mice (*n* = 5 each). The visualization of fluorescent beads movements in the third ventricle allow to approximate the speed of the CSF flux using *in vivo* brain imagery (Figure 3).

Overall, no significant statistical difference in the mean speed between the two groups was found. However, 3/5 animals in the KO group displayed a twofold increase in the mean speed by comparison with the WT group (that shows consistently low speed). However, it should be stressed that we are measuring the bulk flow driven by the arterio-venous pressure gradients and arterial pulsations and that the laminar flow (close to the ventricle wall and therefore dependent upon cilia beating) remains too small to be measured. Furthermore, we believe that the technical caveat of the implantation of the probe and beads injection *in vivo* (blood clot, exact position, size of the probe …) may induce a methodological bias (see Discussion).

## Discussion

The MCH system is involved in a broad spectrum of function through mainly the synaptic release of the peptide(s) and neuronal activity modulation in mammalian brain. However, a growing body of data indicates that MCH may also have non-neuronal function, especially by regulating the activity of more or less specialized peripheral blood mononuclear cells [PBMCs (42)]. The expression of ppMCH and/or MCHR1 genes in pancreatic islets or in adipocytes (43–45) may highlight a metabolism-related function of MCH at the periphery. Nevertheless, such action may not be totally independent of the intracerebral and/or spinal MCH pathway (46). Based on our previous study (1) and the present set of data, we demonstrate a new role for the hypothalamic peptide MCH in modulation of CBF in the ventral part of the third ventricle through activation of ependymal cells.

In combining the laser micro-dissection of ependymal epithelium of the 3V followed by RNA extraction and RT-PCR experiments and *in situ* hybridization data, we demonstrate the presence of MCHR1 transcripts within the epithelium. This is consistent with the data obtained by immunohistochemistry (1) or in a *MCHR1-Cre/tdTomato* mouse model (26).

The optogenetic stimulation of the MCH neurons (through ChETA activation) increased the CBF in the same extend than electrical stimulation did, compared to basal conditions. This confirms also with high temporal precision, the specificity of the response observed in the 3V. Indeed, only ciliated cells lining the ventral 3V that expressed MCHR1 were MCH-sensitive, while those from the dorsal third ventricle or the lateral ventricles were not (1). The spatial specificity of the MCH response adds a new level of complexity to the previously described characteristics of ciliated ependymal cells [orientation, size, beating mode of cilia along the ventricles (47, 48)], and suggests that ciliary beating in cerebral ventricles is fine-tuned to modulate CSF flow in response to metabolic, neurohormonal, and neuroimmune changes. Moreover, our present *in situ* hybridization experiments, previous immunohistochemical data (1) and mapping using *MCHR1-Cre/tdTomato* mouse (26) highly suggested a “cluster-like” distribution of the MCHR1 mRNA and proteins along the 3V. Since adjoining ependymal cells are known to be coupled through GAP junctions (49), it is tempting to speculate that the only few ependymal cells expressing MCHR1 may act as hubs responsible for the effect of MCH neuronal stimulation on the whole epithelium in the 3V.

The results obtained following genetic invalidation and pharmacological inactivation of MCHR1 suggested that the MCH system could exert a tonic positive control on CBF (1). Here, we demonstrate the validity of this hypothesis since the inhibition of MCH neurons activity (through NpHR stimulation) directly affects the CBF. To our knowledge, MCH is the only known molecule exerting a tonic positive effect on cilia beating in the brain. Even more interestingly, this tonic control of MCH on the CBF in the 3V does not seem to be compensated through adaptive mechanisms during development, since the basal CBF in MCHR1-KO mice is also reduced (1).

Even if MCH neurons activity seems to be important in the regulation of cilia beating, we have not yet firmly established whether the communication between MCH neurons and ependymal cells involves a true asymmetric synapse or not. With respect to the anatomical distribution of MCH fibers around the v3V, the communication should more likely involve passing fibers “leaking” MCH close to the epithelium basal pole and/or release of the peptide directly into the CSF. Indeed, MCHR1 immunolabeling was observed at both the apex and the basal poles of ciliated ependymal cells as well as MCH fibers crossing the epithelium. The nature of the contacts between ciliated cells and fibers remains to be addressed using a detailed electronic microscopic analysis.

Genetic ablation of MCHR1 results in an increase in the volume of LVs and 3V (both ventral and dorsal) but no change in 4V as reported previously (1). In order to determine the flow of CSF using a non-invasive method, we have tried to transpose clinical tools (CINE-MRI) to mice. Unfortunately, the main limit of such technique is the speed of the flow. Preliminary experiments indicate that this speed is <10 μm s^−1^, preventing the use of CINE-MRI in the mouse brain (Kober F., Troalen T., and Viola A. CRMBM. Marseille; personal communication).

The most used methods to study the flow of CSF in rodents consist in the injection of tracers (X-rays or MRI contrast agent) *in vivo* (50), or the use of fluorescent micro-beads or china ink *ex vivo* on dissected epithelia (51). Here, we show that it is possible to follow the migration of fluorescent micro-beads through the v3V. Unexpectedly, our data indicate that, in MCHR1-KO mice, the speed of the CSF flow tends to increase as compared to WT littermates, without reaching the statistical significance level. This paradoxical effect could be explained by the Poiseuille’s law which postulates a direct link between the mean speed (*V*) of a viscous liquid (such as CSF) and the radius (*r*) of a small cylinder (such as a ventricle) (*V* = DP*Π*r^4^/8*h* × *l* with *h* = viscosity, DP = difference of pressure between the extremities of the cylinder, *l* = length, and *r* = radius of the cylinder). Indeed, the flow measured at the center of the ventricle would increase when the radius expands. This fits quite well with an enlargement of the ventricle in the KO MCHR1 mice as observed using MRI (1). Another explanation for this discrepancy would be that the optic fiber used for the recording may block the CSF flow in the 3V of WT animals but not in MCHR1-KO (since the volume of this ventricle is enlarged in these animals). Moreover, an increase in the CSF pressure into the ventricles could not be excluded.

At this point, it seems important to dissociate the global flow of CSF, mainly resulting from cardio-respiratory activity, to the laminar flow imputable to cilia beating. This point is of prime importance since it has been shown that MCHR1-KO mice display an increase in heart and respiratory rate (52). As a consequence, this may be responsible for the increase in global CSF flow. Moreover, because the volume of the ventricle is increased in MCHR1-KO mice, according to the Poiseuille’s equation (see above), the speed of the CSF close to the epithelium should be reduced as compared to what is observed in the center of the ventricle. Because of the cilia action, such an effect is reduced and the speed close to epithelial cells is increased. Thus, in MCHR1-KO mice in which the CBF is altered, an increase in the total CSF flow may compensate the local decrease of the flow at the level of cilia. Taking into account all of these parameters, it is not surprising to observe an increase in CSF speed in the 3V.

Our data further suggest that motile ciliated cells of the cerebral ventricles are chemosensory as primary cilia, similar to motile ciliate cells from the airway epithelia (53). Hydrocephalus is one of the features of Bardet–Biedl syndrome (BBS), a genetic disease caused by a mutation in one of several proteins involved in the development of primary cilia, BBS1 being the most frequently affected in humans (54). The characteristics of BBS include ventriculomegaly of the lateral and third ventricles, particularly marked in knockin mice expressing the mutated human BBS1 protein (55). As BBS1 is involved in the trafficking of MCHR1 (54), this ventriculomegaly may be partly due to a defect in MCHR1 expression by ciliated ependymal cells, However, it is worth mentioning that the whole distribution of MCHR1 throughout the brain of BBS mouse models is still lacking. The absence of MCHR1 targeting to primary cilium in BBS models does not seems to affect the ciliogenesis (54). In this context, we found no difference in the morphology of the cilia between WT and MCHR1-KO mice, suggesting that MCHR1 does not play an important role in the development of cilia, but only in the modulation of the CBF, once the cilia are in place (although some compensatory changes could occur during development). This fits quite well with the characterization of MCHR1 mutants in ciliated hREP1 cells and the discovery of a motif in the third intracellular loop that is mandatory for MCHR1 trafficking to the primary cilia but not ciliogenesis. Other GPCR such as, somatostatin 3 receptor (SST3) and serotonin receptor 6 (5-HTR6) are specifically targeted to the primary cilia. Based on the physiological roles of somatostatin, and since new genetically engineered models such as SST3:Cre – cilia GFP mice (56) have been generated, a complete study about SST involvement in CSF and/or CBF regulation should be considered.

In conclusion, this paper and our previous study (1) point to a new role for MCH in maintaining CSF flow and homeostasis in the mouse brain. It is worth noting that the main group of MCH neurons in primitive vertebrates (lampreys) and most fish species (but teleosteans) are located very close (and also projected) to the ventricular surface and could regulate general volume transmission, like in rodents (4, 57). This convergent anatomy could be associated with an ancestral function maintained during evolution. Indeed, MCH neurons could anticipate and initiate the acceleration of CSF circulation, for instance, under conditions of metabolic necessity (glucose withdrawal, fasting, …). The strategic location of the ciliated cells innervated by MCH fibers, at the base of the third cerebral ventricle, could allow them to act as a pump to initiate an increase in CSF flow, providing peptides and other messengers to several brain areas and prolonging the effects of these factors in conjunction with neuronal transmission. Moreover, if the same type of CSF flow regulation exists in humans, this work suggests that the chronic administration of brain penetrating MCHR1 antagonists may have long term side effects due to alterations of CSF flows, limiting the probability of their use as therapeutic agents.

## Authors Contribution

Grégory Conductier conducted and analyzed brain slice imaging experiments (CBF measurements and optogenetics) and participated to writing. Agnès O. Martin, Chrystel Lafont, and Patrice Mollard performed and analyzed the FCFM experiments, and participated in manuscript writing. Pierre-Yves Risold performed laser-captured, RT-PCR and *in situ* hybridization experiments. He participates also to writing. Sonia Jego, Raphaël Lavoie, and Antoine Adamantidis performed vector designing and set up the optogenetics experiments, and participated in manuscript writing. Jean-Louis Nahon acquired funding, designed and analyzed experiments, coordinated collaborations, and participated in manuscript writing and editing.

## Conflict of Interest Statement

The authors declare that the research was conducted in the absence of any commercial or financial relationships that could be construed as a potential conflict of interest.
